# Supplementary material for: Quantifying the nonclassicality of pure dephasing
Source: Nat Commun. 2019 Aug 22;10:3794. doi: 10.1038/s41467-019-11502-4 (PMC6706426; doi:10.1038/s41467-019-11502-4)
Supplement: Supplementary file 1 — Supplementary Information [file 41467_2019_11502_MOESM1_ESM.pdf]

Supplementary Information—Quantifying the nonclassicality of pure dephasing

Chen et al.

Supplementary Note 1. MATHEMATICAL SUPPLEMENTS ON LIE ALGEBRA

In this work, many results rely heavily on the techniques of Lie algebras. For the accessibility to a wide audience in physics, we provide some supplements on Lie algebras.

A. $\mathfrak{u}(n)$ Lie algebra

Since both Hamiltonians $\widehat{H}_\lambda$ and density matrices ρ are Hermitian, it is natural to deal with the problem in the space $\mathfrak{u}(n) = \mathfrak{u}(1) \oplus \mathfrak{su}(n)$, which is spanned by the identity $\{\widehat{I}\}$ and $\{\widehat{L}_m\}_m$ of $n^2 - 1$ traceless Hermitian generators, respectively. Every member Hamiltonian $\widehat{H}_\lambda$ is an element in $\mathfrak{u}(n)$, and can be expressed as a linear combination of the generators

$$\widehat{H}_\lambda = \lambda_0 \widehat{I} + \sum_{m=1}^{n^2-1} \lambda_m \widehat{L}_m = \lambda_0 \widehat{I} + \boldsymbol{\lambda} \cdot \widehat{\mathbf{L}}, \tag{1}$$

where $\lambda_0 \in \mathbb{R}$ and $\boldsymbol{\lambda} = \{\lambda_m\}_m \in \mathbb{R}^{n^2-1}$. Namely, $\lambda = \{\lambda_0, \boldsymbol{\lambda}\} \in \mathbb{R}^{n^2}$ parametrizes the member Hamiltonian $\widehat{H}_\lambda$. Additionally, since $\mathfrak{u}(1)$ commutes with $\mathfrak{su}(n)$ (i.e., $[\mathfrak{u}(1), \mathfrak{su}(n)] = [\lambda_0 \widehat{I}, \boldsymbol{\lambda} \cdot \widehat{\mathbf{L}}] = 0 \ \forall \lambda_0 \in \mathbb{R}, \boldsymbol{\lambda} \in \mathbb{R}^{n^2-1}$), this renders λ_0 playing no role in each single realization of the unitary evolution:

$$\exp[-i\widehat{H}_\lambda t]\rho \exp[i\widehat{H}_\lambda t] = \exp[-i\boldsymbol{\lambda} \cdot \widehat{\mathbf{L}} t]\rho \exp[i\boldsymbol{\lambda} \cdot \widehat{\mathbf{L}} t]. \tag{2}$$

Therefore, we first consider $\mathfrak{su}(n)$, and the space $\mathfrak{u}(1)$ can be easily included latter.

In Lie algebras, $\mathfrak{su}(n)$ itself is a vector space, and equipped with a bilinear Lie bracket

$$[\quad , \quad] : \mathfrak{su}(n) \times \mathfrak{su}(n) \to \mathfrak{su}(n), \tag{3}$$

satisfying the following properties

1. $[\widehat{H}_{\boldsymbol{\lambda}}, \widehat{H}_{\boldsymbol{\lambda}}] = 0$, $\forall \widehat{H}_{\boldsymbol{\lambda}} \in \mathfrak{su}(n)$.
2. $[\widehat{H}_1, [\widehat{H}_2, \widehat{H}_3]] + [\widehat{H}_2, [\widehat{H}_3, \widehat{H}_1]] + [\widehat{H}_3, [\widehat{H}_1, \widehat{H}_2]] = 0$, $\forall \widehat{H}_{\boldsymbol{\lambda}} \in \mathfrak{su}(n)$.

The Lie bracket largely determines the structure of a Lie algebra. This can be understood by applying it to the generators. For $\mathfrak{su}(n)$, the generators satisfy

$$[\widehat{L}_k, \widehat{L}_l] = i2c_{klm}\widehat{L}_m \tag{4}$$

and the c_{klm}'s are called the structure constants, which satisfy

$$c_{klm} = -c_{lkm} = -c_{mlk}, \tag{5}$$

for $\mathfrak{su}(n)$.

B. Representation

To acquire further insight of an abstract Lie algebra, one seminal approach is to link it to another easier one; meanwhile, its algebraic structure can be preserved. This can be achieved by introducing the concepts of homomorphism and representation.

Definition 1 (Lie algebra homomorphism)**.** Let $\mathfrak{L}$ and $\mathfrak{L}'$ be two Lie algebras over the same field $\mathcal{F}$. A linear map $f : \mathfrak{L} \to \mathfrak{L}'$ is a homomorphism if it preserves the Lie brackets:

$$f([\widehat{H}_1, \widehat{H}_2]) = [f(\widehat{H}_1), f(\widehat{H}_2)], \forall \widehat{H}_\lambda \in \mathfrak{L}. \tag{6}$$

A homomorphism is an isomorphism, if it is injective and surjective in the sense of linear maps.

Definition 2 (Representation of a Lie algebra)**.** Let $\mathfrak{L}$ be a Lie algebra over a field $\mathcal{F}$. A representation of $\mathfrak{L}$ is a Lie algebra homomorphism f

$$f : \mathfrak{L} \to \mathfrak{gl}(\mathcal{V}), \tag{7}$$

where $\mathfrak{gl}(\mathcal{V})$ is the general linear algebra of endomorphisms on the vector space $\mathcal{V}$.

Therefore a representation f assigns each $\widehat{H}_\lambda \in \mathfrak{L}$ an endomorphism $f(\widehat{H}_\lambda) : \mathcal{V} \to \mathcal{V}$, depending linearly on $\widehat{H}_\lambda$ and preserving Lie brackets.

C. Adjoint representation

A particularly important representation in the Lie algebra theory is the adjoint representation

$$\mathrm{ad} : \mathfrak{L} \to \mathfrak{gl}(\mathfrak{L}), \tag{8}$$

with

$$\mathrm{ad} : \widehat{H}_\lambda \mapsto \widetilde{H}_\lambda = [\widehat{H}_\lambda, \quad]. \tag{9}$$

In other words, the adjoint representation conceives each $\widehat{H}_\lambda \in \mathfrak{L}$ as an endomorphism $\mathrm{ad}\widehat{H}_\lambda = \widetilde{H}_\lambda$ acting on $\mathfrak{L}$, and its action is implemented by the Lie bracket $\widetilde{H}_\lambda(\widehat{H}_{\lambda'}) = [\widehat{H}_\lambda, \widehat{H}_{\lambda'}]$.

Since a Lie algebra $\mathfrak{L}$ itself is a vector space, this allows one to express each element $\widetilde{H}_\lambda \in \mathfrak{gl}(\mathfrak{L})$ in terms of a matrix with respect to the generator of $\mathfrak{L}$. For $\mathfrak{su}(n)$, the adjoint representation $\widetilde{L}_m$ of each generator $\widehat{L}_m$ is constructed in terms of structure constants c_{klm}.

For example, one generically takes the generators of $\mathfrak{su}(2)$ to be the Pauli matrices, which satisfy the commutation relation

$$[\hat{\sigma}_k, \hat{\sigma}_l] = i2\varepsilon_{klm}\hat{\sigma}_m \tag{10}$$

cyclically. Therefore, the adjoint representation of the Pauli matrices are given by

$$\tilde{\sigma}_x = \begin{bmatrix} 0 & 0 & 0 \\ 0 & 0 & -i2 \\ 0 & i2 & 0 \end{bmatrix}, \tag{11}$$

$$\tilde{\sigma}_y = \begin{bmatrix} 0 & 0 & i2 \\ 0 & 0 & 0 \\ -i2 & 0 & 0 \end{bmatrix}, \tag{12}$$

$$\tilde{\sigma}_z = \begin{bmatrix} 0 & -i2 & 0 \\ i2 & 0 & 0 \\ 0 & 0 & 0 \end{bmatrix}. \tag{13}$$

And any element $\widehat{H}_{\boldsymbol{\lambda}} = \lambda_x \hat{\sigma}_x + \lambda_y \hat{\sigma}_y + \lambda_z \hat{\sigma}_z \in \mathfrak{su}(2)$ has a represention

$$\widetilde{H}_{\boldsymbol{\lambda}} = \boldsymbol{\lambda} \cdot \tilde{\boldsymbol{\sigma}} = \begin{bmatrix} 0 & -i2\lambda_z & i2\lambda_y \\ i2\lambda_z & 0 & -i2\lambda_x \\ -i2\lambda_y & i2\lambda_x & 0 \end{bmatrix}. \tag{14}$$

Since $\mathfrak{u}(1)$ commutes with $\mathfrak{su}(2)$, one can easily extend the representation to the space $\mathfrak{u}(2) = \mathfrak{u}(1) \oplus \mathfrak{su}(2)$, such that for any $\widehat{H}_\lambda = \lambda_0 \widehat{I} + \boldsymbol{\lambda} \cdot \hat{\boldsymbol{\sigma}} \in \mathfrak{u}(2)$, its adjoint representation is explicitly written as

$$\widetilde{H}_\lambda = \left[\begin{array}{c|ccc} 0 & 0 & 0 & 0 \\ \hline 0 & 0 & -i2\lambda_z & i2\lambda_y \\ 0 & i2\lambda_z & 0 & -i2\lambda_x \\ 0 & -i2\lambda_y & i2\lambda_x & 0 \end{array}\right]. \tag{15}$$

Notice that $\widetilde{H}_\lambda$ is independent of λ_0. This reflects the fact that each single unitary evolution in Supplementary Equation (2) has no λ_0 dependence.

Similarly, for the general cases, every $\widehat{H}_\lambda = \lambda_0 \widehat{I} + \boldsymbol{\lambda} \cdot \widehat{\mathbf{L}} \in \mathfrak{u}(n)$ has a representation

$$\widetilde{H}_\lambda = \lambda_0 \widetilde{I} + \boldsymbol{\lambda} \cdot \widetilde{\mathbf{L}} = \left[\begin{array}{c|ccc} 0 & 0 & \cdots & 0 \\ \hline 0 & & & \\ \vdots & & \boldsymbol{\lambda} \cdot \widetilde{\mathbf{L}} & \\ 0 & & & \end{array}\right], \tag{16}$$

which is also independent of λ_0.

Supplementary Note 2. PROOF OF EQ. (3) IN THE MAIN TEXT

Here we present the translation from Eq. (1) into Eq. (3) in the main text. We begin with a useful tool.

Lemma 3. *Let $\widehat{L}$ and $\widehat{M}$ be any elements in the general linear group $\mathrm{GL}(n)$ of $n \times n$ matrices. Then we have the following relation*

$$\exp[\widehat{L}]\widehat{M}\exp[-\widehat{L}] = \sum_{\mu=0}^{\infty} \frac{1}{\mu!} [\widehat{L}, \widehat{M}]_{(\mu)}, \tag{17}$$

where

$$[\widehat{L}, \widehat{M}]_{(0)} = \widehat{M},$$

$$[\widehat{L}, \widehat{M}]_{(1)} = [\widehat{L}, \widehat{M}],$$

$$[\widehat{L}, \widehat{M}]_{(\mu)} = [\widehat{L}, [\widehat{L}, \widehat{M}]_{(\mu-1)}]. \tag{18}$$

This lemma can be proven by straightforwardly expanding $\exp[\pm\widehat{L}]$ with its Taylor series. And an elementary algebra leads to the desired result.

With this lemma, a single realization of the unitary evolution in Eq. (1) in the main text can be rewritten as

$$\exp[-i\widehat{H}_\lambda t]\rho\exp[i\widehat{H}_\lambda t] = \sum_{\mu=0}^{\infty} \frac{(-it)^\mu}{\mu!} [\widehat{H}_\lambda, \rho]_{(\mu)}. \tag{19}$$

One can observe that the right hand side of Supplementary Equation (19) resembles the Taylor series of an exponential. To further recast it into a closed exponential form, we must make use of the adjoint representation of the $\mathfrak{u}(n)$ Lie algebra we have discussed.

A density matrix ρ is also Hermitian and of unital trace; it can be expressed in terms of $\rho = n^{-1}\widehat{I} + \boldsymbol{\rho} \cdot \widehat{\mathbf{L}}$, with $\boldsymbol{\rho} \in \mathbb{R}^{n^2-1}$. One can conceive $\rho = \{n^{-1}, \boldsymbol{\rho}\}$ as an n^2-dimensional column vector, then the action of the commutator $[\widehat{H}_\lambda, \rho]$ can be expressed in terms of conventional matrix multiplication:

$$[\widehat{H}_\lambda, \rho] = \widetilde{H}_\lambda \cdot \rho = \left[\begin{array}{c|ccc} 0 & 0 & \cdots & 0 \\ \hline 0 & & & \\ \vdots & & \boldsymbol{\lambda} \cdot \widetilde{\mathbf{L}} & \\ 0 & & & \end{array}\right] \cdot \left[\begin{array}{c} n^{-1} \\ \hline \\ \boldsymbol{\rho} \\ \\ \end{array}\right], \tag{20}$$

and therefore

$$[\widehat{H}_\lambda, \rho]_{(\mu)} = (\widetilde{H}_\lambda)^\mu \cdot \rho. \tag{21}$$

Consequently, the exponential form of Supplementary Equation (19) follows immediately

$$\begin{aligned} \exp[-i\widehat{H}_\lambda t]\rho\exp[i\widehat{H}_\lambda t] &= \sum_{\mu=0}^{\infty} \frac{(-it)^\mu}{\mu!} (\widetilde{H}_\lambda)^\mu \cdot \rho \\ &= \exp[-i\widetilde{H}_\lambda t] \cdot \rho. \end{aligned} \tag{22}$$

Then, given a time-independent HE $\{(p_\lambda, \widehat{H}_\lambda)\}$, it determines an unital and trace-preserving dynamical linear map $\mathcal{E}_t^{(\widetilde{L})}$ via the Fourier transform on group:

$$\mathcal{E}_t^{(\widetilde{L})} = \int_{\mathbb{R}^{n^2}} p_\lambda e^{-i\widetilde{H}_\lambda t} d\lambda = \int_{\mathbb{R}^{n^2}} p_\lambda e^{-i\lambda\widetilde{L}t} d\lambda, \tag{23}$$

provided $\widetilde{H}_\lambda = \lambda_0 \widetilde{I} + \boldsymbol{\lambda} \cdot \widetilde{\mathbf{L}}$. Notice that $\lambda = \{\lambda_0, \boldsymbol{\lambda}\} \in \mathbb{R}^{n^2}$ and $\widetilde{I} = 0$.

Supplementary Note 3. CONVEXITY OF VARIATIONAL DISTANCE MEASURE

We first note that the set of all legitimate probability distributions is convex since any statistical mixture of probability distributions is again a probability distribution.

Suppose that we are given two pure dephasing dynamics $\mathcal{E}_t^1$ and $\mathcal{E}_t^2$ with $\wp_\lambda^1$ and $\wp_\lambda^2$ being their (quasi-)distributions, respectively. According to our measure of nonclassicality, we have

$$\begin{aligned}&a\mathcal{N}\{\mathcal{E}_t^1\}+(1-a)\mathcal{N}\{\mathcal{E}_t^2\}\\&=a\inf_{p_\lambda}\int_{\mathcal{G}}\frac{1}{2}|\wp_\lambda^1-p_\lambda|d\lambda+(1-a)\inf_{p_\lambda}\int_{\mathcal{G}}\frac{1}{2}|\wp_\lambda^2-p_\lambda|d\lambda.\end{aligned} \tag{24}$$

Suppose that the two infimums are achieved by p_λ^1 and p_λ^2, respectively, we then have

$$\begin{aligned}&a\mathcal{N}\{\mathcal{E}_t^1\}+(1-a)\mathcal{N}\{\mathcal{E}_t^2\}\\&=a\int_{\mathcal{G}}\frac{1}{2}|\wp_\lambda^1-p_\lambda^1|d\lambda+(1-a)\int_{\mathcal{G}}\frac{1}{2}|\wp_\lambda^2-p_\lambda^2|d\lambda\\&\geq\int_{\mathcal{G}}\frac{1}{2}|a\wp_\lambda^1+(1-a)\wp_\lambda^2-[ap_\lambda^1+(1-a)p_\lambda^2]|d\lambda\\&\geq\inf_{p_\lambda}\int_{\mathcal{G}}\frac{1}{2}|a\wp_\lambda^1+(1-a)\wp_\lambda^2-p_\lambda|d\lambda\\&=\mathcal{N}\{a\mathcal{E}_t^1+(1-a)\mathcal{E}_t^2\}.\end{aligned} \tag{25}$$

Therefore, our measure of nonclassicality is convex.

Supplementary Note 4. FINDING THE CHER OF QUBIT PURE DEPHASING

Within a properly chosen basis of its associated Hilbert space, any qubit pure dephasing dynamics can be expressed as

$$\rho_0=\begin{bmatrix}\rho_{\uparrow\uparrow}&\rho_{\uparrow\downarrow}\\\rho_{\downarrow\uparrow}&\rho_{\downarrow\downarrow}\end{bmatrix}\mapsto\mathcal{E}_t\{\rho_0\}=\begin{bmatrix}\rho_{\uparrow\uparrow}&\rho_{\uparrow\downarrow}\phi(t)\\\rho_{\downarrow\uparrow}\phi^*(t)&\rho_{\downarrow\downarrow}\end{bmatrix}. \tag{26}$$

The diagonal elements are constant in time and the off-diagonal elements are governed by the dephasing factor $\phi(t)=\exp[-i\theta(t)-\Phi(t)]$, where $\theta(t)$ ($\Phi(t)$) is a real odd (even) function on time t, respectively, such that $\phi(0)=1$, $|\phi(t)|\leq1$ for all $t\in\mathbb{R}$, and $\phi(-t)=\phi^*(t)$. The first two conditions are for the complete positivity of the dynamics and the last one guarantees that the (quasi-)distribution $\wp$ is a real function.

If we expand ρ in terms of $\rho=2^{-1}\widehat{I}+\boldsymbol{\rho}\cdot\hat{\boldsymbol{\sigma}}$, where $\hat{\boldsymbol{\sigma}}=\{\hat{\sigma}_x,\hat{\sigma}_y,\hat{\sigma}_z\}$ denotes three Pauli matrices, a qubit initial state can be expressed as a four-dimensional column vector

$$\rho_0=\left[\begin{array}{c}1/2\\\hline(\rho_{\uparrow\downarrow}+\rho_{\downarrow\uparrow})/2\\i(\rho_{\uparrow\downarrow}-\rho_{\downarrow\uparrow})/2\\(\rho_{\uparrow\uparrow}-\rho_{\downarrow\downarrow})/2\end{array}\right]. \tag{27}$$

Now we know the action of $\mathcal{E}_t$ on a state, its linear map form $\mathcal{E}_t^{(\tilde{\sigma})}$ can be constructed by applying it to the generators:

1. $\mathcal{E}_t\{\widehat{I}\}=\widehat{I}$.
2. $\mathcal{E}_t\{\hat{\sigma}_x\}=e^{-\Phi(t)}\cos\theta(t)\hat{\sigma}_x+e^{-\Phi(t)}\sin\theta(t)\hat{\sigma}_y$.
3. $\mathcal{E}_t\{\hat{\sigma}_y\}=-e^{-\Phi(t)}\sin\theta(t)\hat{\sigma}_x+e^{-\Phi(t)}\cos\theta(t)\hat{\sigma}_y$.
4. $\mathcal{E}_t\{\hat{\sigma}_z\}=\hat{\sigma}_z$.

We then have the dynamical linear map:

$$\mathcal{E}_t^{(\tilde{\sigma})}=\left[\begin{array}{c|ccc}1&0&0&0\\\hline0&e^{-\Phi(t)}\cos\theta(t)&-e^{-\Phi(t)}\sin\theta(t)&0\\0&e^{-\Phi(t)}\sin\theta(t)&e^{-\Phi(t)}\cos\theta(t)&0\\0&0&0&1\end{array}\right]. \tag{28}$$

On the other hand, the adjoint representation of $\hat{\sigma}_z$ (including the generator $\widehat{I}$ of $\mathfrak{u}(1)$) reads

$$\tilde{\sigma}_z=\left[\begin{array}{c|ccc}0&0&0&0\\\hline0&0&-i2&0\\0&i2&0&0\\0&0&0&0\end{array}\right]. \tag{29}$$

The right-hand side of Eq. (5) in the main text reads

$$\int_{\mathbb{R}}\wp(\omega)e^{-i(\omega\tilde{\sigma}_z/2)t}d\omega=$$
$$\left[\begin{array}{c|ccc}1&0&0&0\\\hline0&\int\wp(\omega)\cos\omega td\omega&-\int\wp(\omega)\sin\omega td\omega&0\\0&\int\wp(\omega)\sin\omega td\omega&\int\wp(\omega)\cos\omega td\omega&0\\0&0&0&1\end{array}\right]. \tag{30}$$

Note that the two matrices (28) and (30) can be simultaneously diagonalized by multiplying

$$X=\left[\begin{array}{c|ccc}1&0&0&0\\\hline0&1&-i&0\\0&1&i&0\\0&0&0&1\end{array}\right],X^{-1}=\left[\begin{array}{c|ccc}1&0&0&0\\\hline0&\frac{1}{2}&\frac{1}{2}&0\\0&\frac{i}{2}&\frac{-i}{2}&0\\0&0&0&1\end{array}\right] \tag{31}$$

from the left and the right, respectively. Namely,

$$X\cdot\mathcal{E}_t^{(\tilde{\sigma})}\cdot X^{-1}=\left[\begin{array}{c|ccc}1&0&0&0\\\hline0&\phi(t)&0&0\\0&0&\phi^*(t)&0\\0&0&0&1\end{array}\right] \tag{32}$$

and

$$\int_{\mathbb{R}} \wp(\omega) X \cdot e^{-i(\omega\tilde{\sigma}_z/2)t} \cdot X^{-1} d\omega =$$

$$\left[\begin{array}{c|ccc} 1 & 0 & 0 & 0 \\ \hline 0 & \int_{\mathbb{R}} \wp(\omega) e^{-i\omega t} d\omega & 0 & 0 \\ 0 & 0 & \int_{\mathbb{R}} \wp(\omega) e^{i\omega t} d\omega & 0 \\ 0 & 0 & 0 & 1 \end{array}\right]. \quad (33)$$

Therefore, the same conclusion

$$\exp[-i\theta(t) - \Phi(t)] = \int_{\mathbb{R}} \wp(\omega) e^{-i\omega t} d\omega \quad (34)$$

is immediately manifest and the conventional inverse Fourier transform leads to the final result.

Supplementary Note 5. DIGONALIZATION AND ITS IMPLICATION

In view of Supplementary Equations (28) and (30), we can easily obtain the result Supplementary Equation (34) without diagonalizing them. Diagonalization seems not necessary. However, the diagonalization provides a deeper insight into the intrinsic algebraic structure. It is essential for a systematic procedure when tackling higher dimensional problems.

To understand the implications of the diagonalization, we recall that, in the adjoint representation $\mathfrak{sl}(\mathfrak{u}(2))$, the Lie algebra $\mathfrak{u}(2)$ plays the role of a vector space with the bases $\{\widehat{I}, \hat{\sigma}_x, \hat{\sigma}_y, \hat{\sigma}_z\}$. The transformation described by X and X^{-1} transforms the bases into $\{\widehat{I}, \hat{\sigma}_+, \hat{\sigma}_-, \hat{\sigma}_z\}$, where $\hat{\sigma}_\pm = (\hat{\sigma}_x \pm i\hat{\sigma}_y)/2$, which are the bases of $\mathfrak{gl}(2) = \mathfrak{u}(1) \oplus \mathfrak{sl}(2)$.

On the other hand, as seen in Supplementary Equation (2), λ_0 is irrelevant in describing the dynamics. We therefore consider only the traceless member Hamiltonian taken from $\mathfrak{H}$ of $\mathfrak{su}(2)$, namely, $\widehat{H}_\omega = \omega\hat{\sigma}_z/2$. The factor 2 is included for later convenience. Its adjoint representation with respect to $\mathfrak{gl}(2)$ basis is obtained by applying $\widetilde{H}_\omega$ on them; namely, $\widetilde{H}_\omega(\hat{\sigma}_\pm) = [\omega\hat{\sigma}_z/2, \hat{\sigma}_\pm] = \pm 1 \cdot \omega\hat{\sigma}_\pm$ and $\widetilde{H}_\omega(\hat{\sigma}_z) = [\omega\hat{\sigma}_z/2, \hat{\sigma}_z] = 0$. Its matrix form is written as

$$\widetilde{H}_\omega = \left[\begin{array}{c|ccc} 0 & 0 & 0 & 0 \\ \hline 0 & \omega & 0 & 0 \\ 0 & 0 & -\omega & 0 \\ 0 & 0 & 0 & 0 \end{array}\right]. \quad (35)$$

The operators $\{\widehat{I}, \hat{\sigma}_+, \hat{\sigma}_-, \hat{\sigma}_z\}$ are the "eigenvectors" of $\widetilde{H}_\omega$ associated with the eigenvalues $\{0, 1, -1, 0\}$, respectively. The eigenvalues ± 1 are therefore referred to as the roots (denoted by $\alpha_{1,2}$) associated to the root spaces span$\{\hat{\sigma}_\pm\}$, spanned by the operators $\hat{\sigma}_\pm$. For higher dimensional systems, the CSA $\mathfrak{H}$ possesses more generators; namely, the member Hamiltonian contains more parameters than a single ω. The roots are no longer real scalars but vectors in an Euclidean space. This can be seen in the following example.

Supplementary Note 6. ROOT SYSTEM

The root space decomposition is a very important tool in the theory of Lie algebras, especially in describing the structure of an abstract Lie algebra, and has many prominent applications in elementary particle physics and gauge field theory. However, to thoroughly understand this technique, we would encounter a divergent bundle of mathematics. This would make it unaccessible to the wide audience in physics. From a practical viewpoint, we instead discuss the following qutrit example, which demonstrates the core concept of the root space decomposition. This is enough for the scope of this work.

A. Qutrit pure dephasing

Consider a qutrit pure dephasing described by

$$\mathcal{E}_t\{\rho_0\} = \begin{bmatrix} \rho_{11} & \rho_{12}\phi_1(t) & \rho_{13}\phi_4(t) \\ \rho_{21}\phi_2(t) & \rho_{22} & \rho_{23}\phi_6(t) \\ \rho_{31}\phi_5(t) & \rho_{32}\phi_7(t) & \rho_{33} \end{bmatrix}. \quad (36)$$

The ordering of the numbering of $\phi_m(t)$ is for the latter convenience. This will become clear in the following discussions. To guarantee the Hermicity of $\rho(t)$, $\phi_1(t) = \phi_2^*(t)$ and so on. Moreover, they satisfy $\phi_m(0) = 1$, $|\phi_m(t)| \leq 1$ for all $t \in \mathbb{R}$, and $\phi_m(-t) = \phi_m^*(t)$.

Inheriting from the Gell-Mann matrices, which form the conventional generators for $\mathfrak{su}(3)$, we define the generators for $\mathfrak{sl}(3)$ as follows:

$$\widehat{K}_1 = \widehat{K}_2^\dagger = \begin{bmatrix} 0 & 1 & 0 \\ 0 & 0 & 0 \\ 0 & 0 & 0 \end{bmatrix}, \widehat{K}_3 = \widehat{L}_3 = \begin{bmatrix} 1 & 0 & 0 \\ 0 & -1 & 0 \\ 0 & 0 & 0 \end{bmatrix},$$

$$\widehat{K}_4 = \widehat{K}_5^\dagger = \begin{bmatrix} 0 & 0 & 1 \\ 0 & 0 & 0 \\ 0 & 0 & 0 \end{bmatrix}, \widehat{K}_6 = \widehat{K}_7^\dagger = \begin{bmatrix} 0 & 0 & 0 \\ 0 & 0 & 1 \\ 0 & 0 & 0 \end{bmatrix},$$

$$\widehat{K}_8 = \widehat{L}_8 = \frac{1}{\sqrt{3}} \begin{bmatrix} 1 & 0 & 0 \\ 0 & 1 & 0 \\ 0 & 0 & -2 \end{bmatrix}. \quad (37)$$

Additionally, $\widehat{K}_0 = \widehat{I}$ is the generator for $\mathfrak{u}(1)$. Then, the dynamical linear map in this basis is a diagonalized

matrix

$$\mathcal{E}_t^{(\widetilde{L})} = \left[\begin{array}{c|cccccccc} 1 & & & & & & & & \\ \hline & \phi_1(t) & & & & & & & \\ & & \phi_2(t) & & & & & & \\ & & & 1 & & & & & \\ & & & & \phi_4(t) & & & & \\ & & & & & \phi_5(t) & & & \\ & & & & & & \phi_6(t) & & \\ & & & & & & & \phi_7(t) & \\ & & & & & & & & 1 \end{array}\right], \tag{38}$$

which is obtained by applying the dynamics on each generator: $\mathcal{E}_t\{\widehat{K}_m\} = \phi_m(t)\widehat{K}_m$.

In general, a 3×3 Hermitian operator is a linear combination of the above 9 generators. However, as seen in Supplementary Equation (2), λ_0 is irrelevant in describing the dynamics. We therefore neglect λ_0 and consider only the traceless member Hamiltonians. Furthermore, since we only consider the elements in $\mathfrak{H}$, the simulating HE is of the form $\{(\wp(\lambda_3, \lambda_8), \widehat{H}_{\boldsymbol{\lambda}})\}_{\lambda_3,\lambda_8}$ with $\widehat{H}_{\boldsymbol{\lambda}} = (\lambda_3 \widehat{L}_3 + \lambda_8 \widehat{L}_8)/2 \in \mathfrak{H}$ and $\boldsymbol{\lambda} = (\lambda_3, \lambda_8) \in \mathbb{R}^2$. By estimating all the commutators $[\widehat{H}_{\boldsymbol{\lambda}}, \widehat{K}_m] = (\boldsymbol{\alpha}_m \cdot \boldsymbol{\lambda})\widehat{K}_m$, we obtain its adjoint representation in the $\mathfrak{gl}(3)$ basis $\widetilde{H}_{\boldsymbol{\lambda}} = (\lambda_3 \widetilde{L}_3 + \lambda_8 \widetilde{L}_8)/2 = \mathrm{diag}\left[\, 0 \,\middle|\, \lambda_3 \;\; -\lambda_3 \;\; 0 \;\; (\lambda_3 + \sqrt{3}\lambda_8)/2 \;\; -(\lambda_3 + \sqrt{3}\lambda_8)/2 \;\; (-\lambda_3 + \sqrt{3}\lambda_8)/2 \;\; -(-\lambda_3 + \sqrt{3}\lambda_8)/2 \;\; 0 \,\right]$, being a diagonal matrix as well.

Finally, from Eq. (4) in the main text, $\mathcal{E}_t^{(\widetilde{L})} = \int_{\mathcal{G}} p_{\lambda} e^{-i\widetilde{H}_\lambda t} d\lambda$, we conclude that the (quasi-)distribution $\wp(\lambda_3, \lambda_8)$ is governed by the following simultaneous Fourier transforms:

$$\begin{cases} \phi_1(t) = \int_{\mathbb{R}^2} \wp(\lambda_3, \lambda_8) e^{-i\lambda_3 t} d\lambda_3 d\lambda_8 \\ \phi_4(t) = \int_{\mathbb{R}^2} \wp(\lambda_3, \lambda_8) e^{-i(\lambda_3 + \sqrt{3}\lambda_8)t/2} d\lambda_3 d\lambda_8 \\ \phi_6(t) = \int_{\mathbb{R}^2} \wp(\lambda_3, \lambda_8) e^{-i(-\lambda_3 + \sqrt{3}\lambda_8)t/2} d\lambda_3 d\lambda_8 \end{cases}. \tag{39}$$

B. Root system of $\mathfrak{su}(3)$

Instead of being engaged in solving the Supplementary Equations (39), we look further insight into its structure in terms of the root system. According to $\widetilde{H}(\lambda_3, \lambda_8)$ above, we can list all the roots of $\mathfrak{su}(3)$:

$$\begin{aligned} \boldsymbol{\alpha}_1 &= -\boldsymbol{\alpha}_2 = (1, 0), \\ \boldsymbol{\alpha}_4 &= -\boldsymbol{\alpha}_5 = \left(\frac{1}{2}, \frac{\sqrt{3}}{2}\right), \\ \boldsymbol{\alpha}_6 &= -\boldsymbol{\alpha}_7 = \left(-\frac{1}{2}, \frac{\sqrt{3}}{2}\right). \end{aligned} \tag{40}$$

They are two dimensional vectors of equal length on the λ_3-λ_8 plane. We plot them in Fig. 2 in the main text.

We can observe that the roots satisfy the following properties:

R1 The roots come in pair, e.g., $\boldsymbol{\alpha}_1$ and $\boldsymbol{\alpha}_2$ are two roots pointing in opposite direction. The three roots $\boldsymbol{\alpha}_1$, $\boldsymbol{\alpha}_4$, and $\boldsymbol{\alpha}_6$ are referred to be positive.

R2 Among the three positive roots, $\boldsymbol{\alpha}_1$ and $\boldsymbol{\alpha}_6$ are simple and $\boldsymbol{\alpha}_4$ is not, since $\boldsymbol{\alpha}_4 = \boldsymbol{\alpha}_1 + \boldsymbol{\alpha}_6$.

R3 All the roots are of equal length and the angle between any two non-pairing roots is either $\pi/3$, $\pi/2$, or $2\pi/3$.

Based on the observations, we can consider $\wp(\lambda_3, \lambda_8)$ as a distribution over the λ_3-λ_8 plane. Now we rewrite

$$\wp(\lambda_3, \lambda_8) d\lambda_3 d\lambda_8 = \wp'(x_1, x_6) dx_1 dx_6 \tag{41}$$

via the change of variables $x_m = \boldsymbol{\alpha}_m \cdot \boldsymbol{\lambda}$, $m = 1, 6$. Note that the Jacobian $\mathrm{Det}[\boldsymbol{\alpha}_1 \;\; \boldsymbol{\alpha}_6]^{-1} = 2/\sqrt{3}$, due to the change of variables has been absorbed into $\wp'(x_1, x_6)$. The first and third lines in Supplementary Equations (39) lead to

$$\begin{cases} \phi_1(t) = \int_{\mathbb{R}} \wp_1(x_1) e^{-ix_1 t} dx_1 \\ \phi_6(t) = \int_{\mathbb{R}} \wp_6(x_6) e^{-ix_6 t} dx_6 \end{cases}. \tag{42}$$

They are the marginals of $\wp$ along the directions $\boldsymbol{\alpha}_1$ and $\boldsymbol{\alpha}_6$, respectively. $\wp_1(x_1)$ and $\wp_6(x_6)$ can be obtained by performing the inverse Fourier transform. Moreover, due to the property R2, the second line in Supplementary Equations (39) describes the correlation between the new random variables x_1 and x_6. If we consider a special case, e.g., $\phi_4(t) = \phi_1(t)\phi_6(t)$, the second equation implies that they are independent:

$$\wp'(x_1, x_6) = \wp_1(x_1)\wp_6(x_6). \tag{43}$$

This finishes solving $\wp$. For the case of correlated random variables, we consider an example of four-dimensions in the following section.

Supplementary Note 7. QUBIT PAIR PURE DEPHASING

We proceed with a non-trivial example in the presence of correlations between random variables. With this example, we can illustrate the intrinsic complexity of the retrieval of (quasi-)distributions.

We consider the extended spin-boson model consisting of a non-interacting qubit pair coupled to a common boson bath. The total Hamiltonian reads

$$\begin{aligned} \widehat{H}_{\mathrm{T}} = & \sum_{j=1,2} \frac{\omega_j}{2} \hat{\sigma}_{z,j} + \sum_{\mathbf{k}} \omega_{\mathbf{k}} \hat{b}_{\mathbf{k}}^\dagger \hat{b}_{\mathbf{k}} \\ & + \sum_{j,\mathbf{k}} \hat{\sigma}_{z,j} \otimes (g_{j,\mathbf{k}} \hat{b}_{\mathbf{k}}^\dagger + g_{j,\mathbf{k}}^* \hat{b}_{\mathbf{k}}). \end{aligned} \tag{44}$$

The whole system evolves unitarily according to the unitary operator (in the interaction picture):

$$\widehat{U}^{\mathrm{I}}(t)=\exp\left[i\sum_{\mathbf{k}}\widehat{Z}_{\mathbf{k}}\widehat{Z}_{\mathbf{k}}^{\dagger}\left(\frac{\omega_{\mathbf{k}}t-\sin\omega_{\mathbf{k}}t}{\omega_{\mathbf{k}}^{2}}\right)\right]\times\exp\left[\sum_{\mathbf{k}}\widehat{Z}_{\mathbf{k}}\alpha_{\mathbf{k}}(t)\hat{b}_{\mathbf{k}}^{\dagger}-\widehat{Z}_{\mathbf{k}}^{\dagger}\alpha_{\mathbf{k}}^{*}(t)\hat{b}_{\mathbf{k}}\right], \quad (45)$$

where $\widehat{Z}_{\mathbf{k}}=\sum_{j=1,2}g_{j,\mathbf{k}}\hat{\sigma}_{z,j}$ and $\alpha_{\mathbf{k}}(t)=-i\int_0^t e^{i\omega_{\mathbf{k}}\tau}d\tau=\left(1-e^{i\omega_{\mathbf{k}}t}\right)/\omega_{\mathbf{k}}$.

For simplicity, we assume that $g_{1,\mathbf{k}}=g_{2,\mathbf{k}}$. Tracing out the boson bath, the qubit pair pure dephasing is described by

$$\mathcal{E}_t\{\rho_0\}=\begin{bmatrix}\rho_{11} & \rho_{12}\phi_1(t) & \rho_{13}\phi_4(t) & \rho_{14}\phi_9(t)\\ \rho_{21}\phi_2(t) & \rho_{22} & \rho_{23}\phi_6(t) & \rho_{24}\phi_{11}(t)\\ \rho_{31}\phi_5(t) & \rho_{32}\phi_7(t) & \rho_{33} & \rho_{34}\phi_{13}(t)\\ \rho_{41}\phi_{10}(t) & \rho_{42}\phi_{12}(t) & \rho_{43}\phi_{14}(t) & \rho_{44}\end{bmatrix}, \quad (46)$$

with dephasing factors

$$\begin{aligned}\phi_1(t)=\phi_4(t)&=\exp[i\theta(t)-\Phi(t)],\\ \phi_6(t)&=1,\\ \phi_9(t)&=\exp[-4\Phi(t)],\\ \phi_{11}(t)=\phi_{13}(t)&=\exp[-i\theta(t)-\Phi(t)],\end{aligned} \quad (47)$$

where

$$\begin{aligned}\theta(t)&=4\int_0^\infty\frac{\mathcal{J}(\omega)}{\omega^2}(\omega t-\sin\omega t)d\omega,\\ \Phi(t)&=4\int_0^\infty\frac{\mathcal{J}(\omega)}{\omega^2}\coth\left(\frac{\hbar\omega}{2k_{\mathrm{B}}T}\right)(1-\cos\omega t)d\omega.\end{aligned} \quad (48)$$

And $\mathcal{J}(\omega)$ is the environmental spectral density function.

Following our procedure, to simulate the qubit pair pure dephasing, we consider the diagonalized member Hamiltonian taken from the CSA $\mathfrak{H}$ of $\mathfrak{su}(4)$:

$$\widehat{H}_{\boldsymbol{\lambda}}=(\lambda_3\widehat{L}_3+\lambda_8\widehat{L}_8+\lambda_{15}\widehat{L}_{15})/2. \quad (49)$$

By estimating all the commutators $[\widehat{H}_{\boldsymbol{\lambda}},\widehat{K}_m]=(\boldsymbol{\alpha}_m\cdot\boldsymbol{\lambda})\widehat{K}_m$, for $m=1,2,\ldots,14$, with $\widehat{K}_m$ being the generators of $\mathfrak{gl}(4)$, we can list all the root vectors of $\mathfrak{su}(4)$:

$$\begin{aligned}\boldsymbol{\alpha}_1&=-\boldsymbol{\alpha}_2=(1,0,0),\\ \boldsymbol{\alpha}_4&=-\boldsymbol{\alpha}_5=\left(\frac{1}{2},\frac{\sqrt{3}}{2},0\right),\\ \boldsymbol{\alpha}_6&=-\boldsymbol{\alpha}_7=\left(-\frac{1}{2},\frac{\sqrt{3}}{2},0\right),\\ \boldsymbol{\alpha}_9&=-\boldsymbol{\alpha}_{10}=\left(\frac{1}{2},\frac{1}{2\sqrt{3}},\sqrt{\frac{2}{3}}\right),\\ \boldsymbol{\alpha}_{11}&=-\boldsymbol{\alpha}_{12}=\left(-\frac{1}{2},\frac{1}{2\sqrt{3}},\sqrt{\frac{2}{3}}\right),\\ \boldsymbol{\alpha}_{13}&=-\boldsymbol{\alpha}_{14}=\left(0,-\frac{1}{\sqrt{3}},\sqrt{\frac{2}{3}}\right).\end{aligned} \quad (50)$$

Note that the roots $\boldsymbol{\alpha}_1,\ldots,\boldsymbol{\alpha}_7$, lying on the λ_3-λ_8 plane, are the same as those of $\mathfrak{su}(3)$. The root system of $\mathfrak{su}(4)$ is even more complicated. For visual clarity, we only show six positive roots in Fig. 3b in the main text. Moreover, among the six positive roots, $\boldsymbol{\alpha}_1$, $\boldsymbol{\alpha}_6$, and $\boldsymbol{\alpha}_{13}$ are simple because other positive roots can be obtained by combining them, e.g., $\boldsymbol{\alpha}_9=\boldsymbol{\alpha}_1+\boldsymbol{\alpha}_6+\boldsymbol{\alpha}_{13}$ and $\boldsymbol{\alpha}_{11}=\boldsymbol{\alpha}_6+\boldsymbol{\alpha}_{13}$.

From the equation $\mathcal{E}_t^{(\widetilde{L})}=\int_{\mathbb{R}^3}p(\boldsymbol{\lambda})e^{-i\widetilde{H}_{\boldsymbol{\lambda}}t}d^3\boldsymbol{\lambda}$, the (quasi-)distribution $\wp(\boldsymbol{\lambda})$, over $\mathbb{R}^3$ space, is governed by six simultaneous Fourier transforms:

$$\phi_m(t)=\int_{\mathbb{R}^3}\wp(\boldsymbol{\lambda})e^{-i(\boldsymbol{\alpha}_m\cdot\boldsymbol{\lambda})t}d^3\boldsymbol{\lambda},\ m=1,4,6,9,11,13. \quad (51)$$

Generically, the three random variables are correlated. To solve the correlated $\wp$, we therefore perform the change of variables $x_m=\boldsymbol{\alpha}_m\cdot\boldsymbol{\lambda}$, $m=1,6,13$, because they are simple and can be used to expand the other roots, and we rewrite

$$\wp(\lambda_3,\lambda_8,\lambda_{15})d\lambda_3d\lambda_8d\lambda_{15}=\wp'(x_1,x_6,x_{13})dx_1dx_6dx_{13}. \quad (52)$$

Note that the Jacobian $\mathrm{Det}[\boldsymbol{\alpha}_1\ \boldsymbol{\alpha}_6\ \boldsymbol{\alpha}_{13}]^{-1}=\sqrt{2}$ due to the change of variables has been absorbed into $\wp'$. Then, the three axes of $\wp'$ are defined by the three simple roots.

Additionally, since $\phi_6(t)=1$, we can observe the following correspondence between the root vectors and the dephasing factors:

$$\begin{aligned}\boldsymbol{\alpha}_1+\boldsymbol{\alpha}_6=\boldsymbol{\alpha}_4&\leftrightarrow\phi_1(t)\phi_6(t)=\phi_4(t),\\ \boldsymbol{\alpha}_6+\boldsymbol{\alpha}_{13}=\boldsymbol{\alpha}_{11}&\leftrightarrow\phi_6(t)\phi_{13}(t)=\phi_{11}(t).\end{aligned} \quad (53)$$

This implies that $\wp'=\wp_6(x_6)\wp_{1,13}(x_1,x_{13})$ is separated into two parties and they can be determined according

to the set of equations:

$$\phi_1(t) = \int_{\mathbb{R}} \wp_1(x_1) e^{-ix_1 t} dx_1,$$
$$\phi_{13}(t) = \int_{\mathbb{R}} \wp_{13}(x_{13}) e^{-ix_{13} t} dx_{13},$$
$$\phi_9(t) = \int_{\mathbb{R}^2} \wp_{1,13}(x_1, x_{13}) e^{-ix_1 t} e^{-ix_{13} t} dx_1 dx_{13},$$
$$1 = \int_{\mathbb{R}} \wp_6(x_6) e^{-ix_6 t} dx_6. \quad (54)$$

The first and second line specify the marginals of $\wp_{1,13}(x_1, x_{13})$ along the directions $\boldsymbol{\alpha}_1$ and $\boldsymbol{\alpha}_{13}$, respectively; meanwhile, the third line describes the correlation between them. The last line immediately leads to the result $\wp_6(x_6) = \delta(x_6)$.

Consider the Ohmic spectral density $\mathcal{J}(\omega) = \omega \exp(-\omega/\omega_c)$ in the zero-temperature limit, the dephasing factors can be calculated explicitly:

$$\phi_1(t) = e^{i\theta(t) - \Phi(t)} = \frac{\exp[i(4\omega_c t - 4\arctan(\omega_c t))]}{(1 + \omega_c^2 t^2)^2},$$
$$\phi_{13}(t) = e^{-i\theta(t) - \Phi(t)} = \frac{\exp[-i(4\omega_c t - 4\arctan(\omega_c t))]}{(1 + \omega_c^2 t^2)^2},$$
$$\phi_9(t) = e^{-4\Phi(t)} = \frac{1}{(1 + \omega_c^2 t^2)^8}. \quad (55)$$

The two marginals are easily obtained by the conventional Fourier transform

$$\wp_1(x_1) = \frac{1}{2\pi} \int_{-\infty}^{\infty} \phi_1(t) e^{ix_1 t} dt = \begin{cases} \frac{1}{6\omega_c^4}(x_1 + 4\omega_c)^3 e^{-\frac{x_1 + 4\omega_c}{\omega_c}} & , x_1 \geq -4\omega_c \\ 0 & , x_1 < -4\omega_c \end{cases} \quad (56)$$

and

$$\wp_{13}(x_{13}) = \frac{1}{2\pi} \int_{-\infty}^{\infty} \phi_{13}(t) e^{ix_{13} t} dt = \begin{cases} 0 & , x_{13} > 4\omega_c \\ \frac{-1}{6\omega_c^4}(x_{13} - 4\omega_c)^3 e^{\frac{x_1 - 4\omega_c}{\omega_c}} & , x_{13} \leq 4\omega_c \end{cases}. \quad (57)$$

However, since $\phi_1(t)\phi_{13}(t) \neq \phi_9(t)$, this implies that the two random variables x_1 and x_{13} are correlated and $\wp_1(x_1)\wp_{13}(x_{13}) \neq \wp_{1,13}(x_1, x_{13})$. A difficulty in solving $\wp_{1,13}$ lies in the fact that, in the third line of Supplementary Equations (54), there are two random variables, but they are accompanied with the same time variable t.

Interestingly, this can easily be solved by a simple ansatz. Let

$$\theta(t_1 - t_{13}) = 4\omega_c(t_1 - t_{13}) - 4\arctan[\omega_c(t_1 - t_{13})],$$
$$\tau(t_1, t_{13}) = 2^{\frac{8t_1 t_{13}}{(t_1 + t_{13})^2}},$$
$$\Psi(t_1, t_{13}) = 2\tau(t_1, t_{13}) \ln\left[1 + \omega_c^2 \frac{(t_1 + t_{13})^2}{\tau(t_1, t_{13})}\right]. \quad (58)$$

One can observe that $\tau(t, 0) = \tau(0, t) = 1$ and $\tau(t, t) = 4$, then

$$\exp[i\theta(t_1 - t_{13}) - \Psi(t_1, t_{13})] = \int\int_{-\infty}^{\infty} \wp_{1,13}(x_1, x_{13}) e^{-ix_1 t_1} e^{-ix_{13} t_{13}} dx_1 dx_{13} \quad (59)$$

simultaneously recovers the first three lines in Supplementary Equations (54); namely, $\{t_1 = t, t_{13} = 0\}$ recovers the first line, $\{t_1 = 0, t_{13} = t\}$ recovers the second line, and $\{t_1 = t, t_{13} = t\}$ recovers the third line. Meanwhile, it is a conventional two-dimensional Fourier transform with distinct time variables t_1 and t_{13}. Therefore, $\wp_{1,13}$ can be solved by

$$\wp_{1,13}(x_1, x_{13}) = \frac{1}{4\pi^2} \times \int\int_{-\infty}^{\infty} e^{i\theta(t_1 - t_{13}) - \Psi(t_1, t_{13})} e^{ix_1 t_1} e^{ix_{13} t_{13}} dt_1 dt_{13}. \quad (60)$$

This concludes the solution of Supplementary Equations (54). The numerical result is shown in Fig. 3c in the main text.

Supplementary Note 8. PROOF OF EXISTENCE AND UNIQUENESS

After introducing our procedure, we now show the proof of the existence and uniqueness of the CHER for pure dephasing. Since we deal with (quasi-)distribution functions $\wp(\lambda)$, which are real [$\wp(\lambda) \in \mathbb{R}$], normalized [$\int \wp(\lambda) d\lambda = 1$], but not necessarily positive, we start with the $L^1(\mathcal{G})$ space consisting of real functions defined on a locally compact and ablian group $\mathcal{G}$ (generated by CSA $\mathfrak{H}$) such that their absolute values are Lebesgue integrable. Note that the $L^1(\mathcal{G})$ forms a vector space and is a super set of all (quasi-)distributions. Conversely, an arbitrary element $f \in L^1(\mathcal{G})$ may not necessarily be normalized.

Besides the addition in $L^1(\mathcal{G})$, we further define a binary operation, the "multiplication" $* : L^1(\mathcal{G}) \times L^1(\mathcal{G}) \to L^1(\mathcal{G})$, in terms of the convolution:

$$h(\lambda) = (f * g)(\lambda) = \int_{\mathcal{G}} f(\lambda - \xi) g(\xi) d\xi \in L^1(\mathcal{G}), \ \forall\ f, g \in L^1(\mathcal{G}). \quad (61)$$

Equipped with this multiplication, $L^1(\mathcal{G})$ forms a Banach algebra. We assign the delta function $\delta(\lambda)$ the role of multiplicative identity in the sense that

$$(f * \delta)(\lambda) = (\delta * f)(\lambda) = f(\lambda), \ \forall\ f \in L^1(\mathcal{G}). \quad (62)$$

On the other hand, consider the adjoint representation $\mathfrak{gl}(\mathfrak{u}(n))$ in the basis of $\mathfrak{gl}(n)$, we define a subset $\mathcal{D} \subset \mathfrak{gl}(\mathfrak{u}(n))$ consisting of diagonalized maps such that their entries satisfy the conditions:

1. Every $\mathcal{E}_t^{(\widetilde{L})} \in \mathcal{D}$ is diagonalized.

2. The entry of $\mathcal{E}_t^{(\widetilde{L})}$ corresponding to $\widehat{I}$ is a real constant $A \in \mathbb{R}$.

3. The entries of $\mathcal{E}_t^{(\widetilde{L})}$ corresponding to opposite root spaces are complex conjugate to each other, $\phi_{-\vec{\alpha}}(t) = \phi^*_{\vec{\alpha}}(t)$, and satisfy $\phi_{\vec{\alpha}}(0) = 1$ and $\phi_{\vec{\alpha}}(-t) = \phi^*_{\vec{\alpha}}(t)$.

4. The entries of $\mathcal{E}_t^{(\widetilde{L})}$ corresponding to the $\mathfrak{H}$ of $\mathfrak{sl}(n)$ are 1.

Note that $\mathcal{D}$ forms an abelian group and the set of all CPTP pure dephasing dynamical maps is its subset.

After identifying the algebraic structures, the Fourier transform $\mathcal{E}_t^{(\widetilde{L})} = \int_{\mathcal{G}} \wp(\lambda) e^{-i\lambda \widetilde{L} t} d\lambda$ can be conceived as a map: $\wp(\lambda) \mapsto \mathcal{E}_t^{(\widetilde{L})}$. Then, given $\widetilde{L}_m \in \mathfrak{H}$, the Fourier transform is an isomorphism from $L^1(\mathcal{G})$ to $\mathcal{D}$. This is stated in the following critical Lemma:

Lemma 4. *The Fourier transform with generators $\widetilde{L}_m$ taken from the CSA $\mathfrak{H}$ of $\mathfrak{gl}(\mathfrak{u}(n))$ is an isomorphism from $L^1(\mathcal{G})$ to $\mathcal{D}$.*

Proof. Suppose that f and g are two elements of $L^1(\mathcal{G})$, and $\mathcal{E}_f^{(\widetilde{L})}$ and $\mathcal{E}_g^{(\widetilde{L})}$ are their Fourier transform, with generators $\widetilde{L}_m \in \mathfrak{H}$, respectively. Let $h = f * g$, then

$$\begin{aligned} \mathcal{E}_h^{(\widetilde{L})} &= \int_{\mathcal{G}} h(\lambda) e^{-i\lambda \widetilde{L} t} d\lambda \\ &= \int_{\mathcal{G}} (f * g)(\lambda) e^{-i\lambda \widetilde{L} t} d\lambda \\ &= \int_{\mathcal{G}} \int_{\mathcal{G}} f(\lambda - \xi) e^{-i\lambda \widetilde{L} t} g(\xi) d\xi d\lambda \\ &= \int_{\mathcal{G}} f(\lambda - \xi) e^{-i(\lambda - \xi)\widetilde{L} t} d\lambda \int_{\mathcal{G}} g(\xi) e^{-i\xi \widetilde{L} t} d\xi. \end{aligned} \tag{63}$$

The last line is valid with the following two properties. First, for the generators $\widetilde{L}_m \in \mathfrak{H}$, they commute with each other. Otherwise, we must appeal to the BCH formula. Second, since $\mathcal{G}$ is an abelian group and λ runs over all group elements, the rearrangement lemma guarantees that $\lambda' = \lambda - \xi$ is also the case. Then, we have

$$\mathcal{E}_h^{(\widetilde{L})} = \mathcal{E}_f^{(\widetilde{L})} \mathcal{E}_g^{(\widetilde{L})}. \tag{64}$$

Therefore, the Fourier transform is a multiplicative homomorphism from $L^1(\mathcal{G})$ to $\mathcal{D}$.

In addition, it is obvious that

$$\mathrm{id}^{(\widetilde{L})} = \int_{\mathcal{G}} \delta(\lambda) e^{-i\lambda \widetilde{L} t} d\lambda, \ \forall\, t \in \mathbb{R}. \tag{65}$$

This means that the multiplicative identity $\delta(\lambda)$ in $L^1(\mathcal{G})$ is mapped to the identity map $\mathrm{id}^{(\widetilde{L})}$ in $\mathcal{D}$, with all diagonal entries being 1. Additionally, $\delta(\lambda)$ is the only element in the kernel of the Fourier transform with $\widetilde{L}_m \in \mathfrak{H}$. Namely, $\delta(\lambda)$ is the only solution satisfying Supplementary Equation (65). This can easily be seen from our procedure Eq. (8) in the main text. Consequently, this proves our results that the Fourier transform with $\widetilde{L}_m \in \mathfrak{H}$ is an isomorphism. ■

This lemma ensures the one-one correspondence between $L^1(\mathcal{G})$ and $\mathcal{D}$. Moreover, a CPTP pure dephasing is an element in $\mathcal{D}$ with $A = 1$; this is equivalent to a normalized $\wp(\lambda)$. This proves the existence and uniqueness of the simulating HE with diagonalized member Hamiltonians for CPTP pure dephasing.

Supplementary Note 9. SIMULATING THE NOISE IN THE S-T_0 PURE DEPHASING EXPERIMENT

Experiments inevitably suffer from the disturbances caused by the fluctuations of the surrounding environment or the imperfection of the measurements. Therefore, the experimentally measured raw data may potentially deviate from the theoretical prediction of an idealized model.

The prototype of our theoretical model is the S-T_0 qubit in a gate-defined double-quantum-dot device, fabricated in a GaAs/AlGaAs heterostructure. The reported spin relaxation time (T_1) in such material can approach several milliseconds, while the time-averaged dephasing time (T_2^*) is on the time scale of tens of nanoseconds. Therefore the qubit dynamics can be well approximated as pure dephasing.

In the quantum state tomography experiment, the S-T_0 qubit state is constructed by projective measurements onto the three axes of the Bloch sphere defined as $|\mathrm{X}\rangle = (|\mathrm{S}\rangle + |\mathrm{T}_0\rangle)/\sqrt{2} = |\uparrow\downarrow\rangle$, $|\mathrm{Y}\rangle = (|\mathrm{S}\rangle - i|\mathrm{T}_0\rangle)/\sqrt{2}$, and $|\mathrm{Z}\rangle = |\mathrm{S}\rangle = (|\uparrow\downarrow\rangle - |\downarrow\uparrow\rangle)/\sqrt{2}$, and measuring the corresponding return probabilities $P_{|j\rangle}(\tau_\mathrm{s})$, $j = \mathrm{X}, \mathrm{Y}, \mathrm{Z}$, at different free induction decay times τ_s, as indicated by the blue curves in Supplementary Figure 1a. Once all the $P_{|j\rangle}(\tau_\mathrm{s})$ are given, we can construct the density matrix $\rho(\tau_\mathrm{s}) = [\widehat{I} + \sum_{j=\mathrm{X},\mathrm{Y},\mathrm{Z}} r_j(\tau_\mathrm{s}) \hat{\sigma}_j]/2$ with the trajectories $\mathbf{r}(\tau_\mathrm{s}) = \{r_\mathrm{X}(\tau_\mathrm{s}), r_\mathrm{Y}(\tau_\mathrm{s}), r_\mathrm{Z}(\tau_\mathrm{s})\}$ in the Bloch sphere determined by

$$r_j(\tau_\mathrm{s}) = 2P_{|j\rangle}(\tau_\mathrm{s}) - 1,\ j = \mathrm{X}, \mathrm{Y}, \mathrm{Z}. \tag{66}$$

Then we can apply the analysis explained in the main text and the Methods section.

We complement our theoretical simulation of the S-T_0 qubit pure dephasing by including noise effects in terms of statistical fluctuations. The detailed simulation of the noise effects, as well as our complete analysis, are outlined step by step in the following, and schematically in Supplementary Figure 1.

Step 1 Based on the theoretically simulation (blue curves), the brown points in Supplementary Figure 1a are randomly offset vertically, following a

Gaussian noise distribution with standard deviation 0.05. Then the brown curves fitting the noisy data points simulate the noisy experimental measurement.

Step 2 Depict the trajectories in the Bloch sphere according to Supplementary Equation (66) and identify the angle Ω between the axis of rotation (blue vector), i.e., the normal vector of the blue dephasing disk, and $|\mathrm{S}\rangle$-axis, as shown in Supplementary Figure 1b.

Step 3 Perform a unitary rotation $\widehat{R}_\Omega \rho(\tau_\mathrm{s}) \widehat{R}_\Omega^\dagger$, with $\widehat{R}_\Omega = \exp[i\Omega\hat{\sigma}_\mathrm{Y}/2]$, as shown in Supplementary Figure 1c. This recovers the standard form Eq. (2) in the main text.

Step 4 For the idealized (blue) trajectory, our procedure is directly applicable and leads to the numerical result $\wp(\omega)$ in Supplementary Figure 1d. For the noisy (brown) trajectory, we first project it onto the dephasing disk. Then we can again apply our procedure.

Step 5 Estimate the nonclassicality $\mathcal{N}$ according to Eq. (4) in the main text, and repeatedly perform the noise simulation. This way, we can obtain a series of fluctuating nonclassicality $\mathcal{N}$ values. By taking the mean value and the standard deviation of the $\mathcal{N}$ series, we obtain the average nonclassicality $\mathcal{N}$ (brown points) and the brown error bars shown in Fig. 4d in the main text.

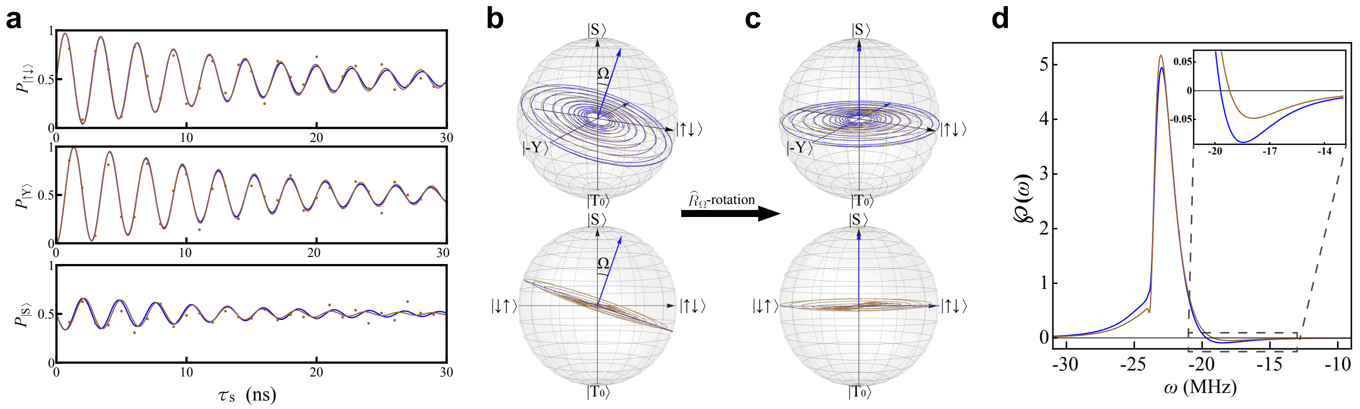

Supplementary Figure 1. **Schematic illustration of our numerical analysis of the noisy S-T$_0$ qubit pure dephasing.** **a** The blue curves stem from the theoretical model. The brown points are randomly offset vertically from the blue curves, following a Gaussian noise distribution with standard deviation 0.05. Then the brown curves fitting the noisy data points simulate the noisy experimental measurement. **b** After all the $P_{|j\rangle}(\tau_\mathrm{s})$ being given, we can depict the trajectories in a Bloch sphere and the dynamics are therefore explicitly visualized. The theoretical (blue) trajectory defines a clear dephasing disk. Its normal vector and the angle Ω between the $|\mathrm{S}\rangle$-axis can be identified. However, the noisy (brown) trajectory does not perfectly attach to the dephasing disk. The two panels are shown from different viewing angles. **c** According to the normal vector identified in (**b**), a unitary rotation $\widehat{R}_\Omega$ recovers the standard form in Eq. (2) in the main text. The two panels are shown from different viewing angles. **d** Applying our procedure explained in the main text, we can numerically recover the desired $\wp(\omega)$. Finally, repeatedly performing the noise simulation leads to a series of fluctuating $\mathcal{N}$ values. Then taking the mean value and the standard deviation, we obtain the average nonclassicality $\mathcal{N}$ (brown points) and the brown error bars shown in Fig. 4**d** in the main text.
